# A Novel Detachable, Reusable, and Versatile Acoustic Tweezer Manipulation Platform for Biochemical Analysis and Detection Systems

**DOI:** 10.3390/bios12121179

**Published:** 2022-12-18

**Authors:** Yukai Liu, Miaomiao Ji, Yichi Zhang, Xiaojun Qiao, Nanxin Yu, Chenxi Ding, Lingxiao Yang, Rui Feng, Xiujian Chou, Wenping Geng

**Affiliations:** 1 Science and Technology on Electronic Test and Measurement Laboratory, North University of China, Taiyuan 030051, China; 2 Key Laboratory of Instrumentation Science &Dynamic Measurement, North University of China, Taiyuan 030051, China

**Keywords:** acoustic tweezer, manipulation platform, detachability, versatility, reusability

## Abstract

Multifunctional, integrated, and reusable operating platforms are highly sought after in biochemical analysis and detection systems. In this study, we demonstrated a novel detachable, reusable acoustic tweezer manipulation platform that is flexible and versatile. The free interchangeability of different detachable microchannel devices on the acoustic tweezer platform was achieved by adding a waveguide layer (glass) and a coupling layer (polydimethylsiloxane (PDMS) polymer film). We designed and demonstrated the detachable multifunctional acoustic tweezer platform with three cell manipulation capabilities. In Demo I, the detachable acoustic tweezer platform is demonstrated to have the capability for parallel processing and enrichment of the sample. In Demo II, the detachable acoustic tweezer platform with capability for precise cell alignment is demonstrated. In Demo III, it was demonstrated that the detachable acoustic tweezer platform has the capability for the separation and purification of cells. Through experiments, our acoustic tweezer platform has good acoustic retention ability, reusability, and stability. More capabilities can be expanded in the future. It provides a simple, economical, and multifunctional reusable operating platform solution for biochemical analysis and detection systems.

## 1. Introduction

In the biochemical analysis and detection system, the multifunctional integrated repeatable operating platform is always the goal scholars are keen to pursue [1,2]. For example, several key steps in single cell analysis (such as cell capture [3], arrangement [4], separation [5], and other operations) are integrated on one platform to realize free switching of multiple functions. There are various methods of cell manipulation based on microfluidics, mainly divided into passive techniques [6,7,8] and active techniques [9,10,11]. The latter is widely used because it allows for more precise manipulation of the cells. Active technology generally manipulates cells through external force fields such as the electric field [12,13], magnetic field [14,15] and acoustic field [16,17]. Acoustic manipulation technology has attracted extensive attention and research because of its advantages of non-contact, no damage, no marking, and easy manipulation. There are several acoustic manipulations, namely, traveling surface acoustic wave (TSAW) [18,19], surface standing wave (SSAW) [20,21], and bulk acoustic waves (BAW) [22,23]. The SSAW-based acoustic tweezer technology is widely favored by researchers because of its more flexible and diverse manipulation of cells.

Currently, most acoustic tweezer microfluidic devices are made by irreversibly bonding the microchannel to the substrate, so they are disposable [24,25]. The price of substrate materials (such as lithium niobate (LiNbO_3_)) and the sputtering metals (such as gold) remain expensive, posing significant cost challenges for disposable chips. In the biochemical analysis, different chips need to be replaced to avoid cross-infection, and the high cost will likely limit its use. Secondly, in the current research, most acoustic tweezer devices have a single function [20], which is fundamental because the devices are manufactured at one time. Zhichao Ma et al. [26] proposed a detachable acoustic flow system. The PDMS microchannel was combined with the plasma-treated PDMS microcolumn layer to form a disposable microchannel, which was then placed on an interdigital transducer (IDT). They used TSAW instead of SSAW to achieve particle sorting, but this work gave us a way to recycle (which can be reused) the surface acoustic wave (SAW) device. However, the use of a PDMS soft film as the coupling layer of leaky SAW directly may possibly cause a nodal error, which makes it difficult to form stable standing waves. In addition, the thicker PDMS layer as the microchannel sealing layer will increase the acoustic loss. Junping Duan et al. [19] used a thin PDMS film as the sealing layer. The bottom of the microchannel coupled in this way may be uneven, and there may be leakage, deformation, and other problems. In their works [19,27], the microchannel had to be replaced with a new one after several uses due to the reduced adhesion of the underside seal. Jingui Qian et al. [27] added a hard substrate (silicon) on previous basis to improve coupling efficiency. Polymer films were added between the SAW device and the silicon layer to achieve reliable fixation of the detachable acoustic flow device, and the stability was improved. The opacity of the silicon substrate makes the subsequent detection somewhat limited (e.g., inverted microscope observation).

While many researchers have demonstrated the recyclable and reusable capability of SAW devices, they have yet to show the potential of such chips for other scenarios. Unlike in other work, we demonstrated a new detachable acoustic tweezer manipulation platform that is flexible and versatile. With this platform, the free interchangeability of multiple microchannel chips is realized, and the different functions of the corresponding chips are demonstrated. Thin sheet glass was selected as a high-quality acoustic transmission waveguide layer. On the one hand, the smoothness of the bottom of the microchannel is ensured. On the other hand, the high light transmittance is ensured and the anechoic angle area is smaller. A polymer film (PDMS) serves as a coupling agent below the glass layer to balance the acoustic coupling efficiency, stability, and reusability. When the adhesion between the PDMS coupling layer and the substrate decreases, the new PDMS coupling layer can be replaced without abandoning the microchannel. The soft contact between the substrates protects the transducer from mechanical damage in the case of multiple replacements. The bidirectionally propagated two columns of TSAW are transmitted through the polymer film coupling layer to the acoustic waveguide layer (glass), forming a stable standing wave. Three microchannels with different structures are designed to test the performance of the detachable acoustic tweezer manipulation platform. The capture, arrangement, and separation of particles are achieved by changing different microchannels. During the test, the acoustic tweezer device is always fixed. Switching between different operation functions only requires replacing the suitable overlaying channel, and the transparent waveguide layer creates conditions for many detection scenarios (e.g., using an upright or inverted microscope to observe). This novel detachable acoustic tweezer manipulation platform enables the recovery and reuse of acoustic tweezer components and demonstrates the possibility of realizing more manipulation functions in the biochemical analysis. It provides a simple and cost-effective solution for particle or cell manipulation in biomedical applications, and more capabilities can be expanded in the future. We believe that our design has the potential to inspire the design of other detachable acoustofluidic devices for more lab-on-a-chip applications.

## 2. Materials and Methods

### 2.1. Working Mechanism

**Figure 1:**
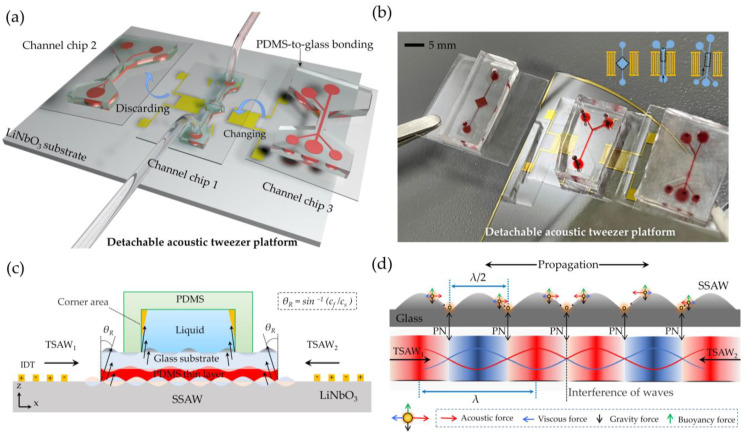
Detachable acoustic tweezer manipulation platform: (**a**) working diagram of the platform, (**b**) photograph of the platform, (**c**) device composition on the cross-section and SSAW radiation schematic, and (**d**) pressure nodes and force analysis.

The working diagram of the whole device is shown in Figure 1a. The proposed particle manipulation platform consists of a freely moving microchannel and an SSAW device. Different particle manipulation functions can be realized by replacing detachable microchannels with different structures. A picture of the platform is shown in Figure 1b. As shown in Figure 1c, the detachable chip consists of a PDMS microchannel bonded to a glass sheet with a PDMS thin layer on the underside. The glass can be used as the waveguide layer because of its good acoustic transmission performance. The soft contact between the chip and the platform can be realized by sticking PDMS soft film under the glass. In this way, the transparent glass and the microchannel layer above it can be reliably fixed on the acoustic tweezer platform, and the mechanical damage caused by the acoustic tweezer devices after repeated use can be effectively reduced. Here, 128°Y-X LiNbO_3_ was selected for the piezoelectric substrate. This type of cutting has the highest electromechanical coupling coefficient and the lowest insertion loss, and has thus become the most popular and widely accepted orientation for applications requiring Rayleigh waves [28]. When a radio frequency signal (RF) is applied to the symmetrically patterned IDTs on a piezoelectric substrate, the bidirectionally propagated TSAWs can be externed by two transducers. When SAW enters another medium, it will radiate at the interface at a Rayleigh angle of *θ_R_* = sin^−1^(*C_f_/C_S_*) [29], producing a leaky SAW. The radiation process passes through three interfaces, LiNbO_3_–PDMS, PDMS–glass, and glass–water, and the Rayleigh angle is *θ_R1_* ≈ 39.3°, *θ_R2_* = 0°, and *θ_R3_* ≈ 15.3°, respectively. The high acoustic speed in the waveguide layer (glass) causes acoustic waves to radiate at an extremely small angle (15.3°) as they enter the fluid, resulting in an almost negligible corner region (see orange area in Figure 1c). The leaky SAW formed a stable SSAW field above the waveguide layer through the phase-length interference, with periodic and stable pressure nodes, as shown in Figure 1d.

The particles are subjected to four forces in the microchannel, namely, acoustic radiation force *F_SSAW_*, stokes resistance *F_drag_*, buoyancy, and gravity. Where buoyancy and gravity maintain a balance along the vertical axis due to the similar density of the microsphere and deionized water, as shown in Figure 1d. When acoustic waves travel across the surface of LiNbO_3_, *F_SSAW_* can be expressed as [30]:

(1) $$F_{SSAW}=\left(\frac{\pi P_{0}^{2}V_{P}\beta_{f}}{2\lambda }\right)\phi \left(\beta ,\rho \right)sin\left(\frac{4\pi }{\lambda }x\right)$$

where

(2) $$\phi \left(\beta ,\rho \right)=\frac{5\rho_{p}-2\rho_{f}}{2\rho_{p}+\rho_{f}}-\frac{\beta_{p}}{\beta_{f}}$$

In Equation (1), *P*_0_ represents the pressure of the SSAW field. *V_p_* is the volume of the microsphere. *β* = 1/(*ρc*)^2^ is compressibility, where *c* and *ρ* are acoustic speed and density, respectively. When the same medium particle (e.g., PS particle, $\phi_{PS}>0$) is placed in a determined acoustic field, *P_0_*, λ, and *β* = 1/(*ρc*)^2^ are determined. At this point, *F_SSAW_* is only a function of the volume *Vp* and *sin(x)*. Therefore, particles with a larger volume *Vp* are subjected to a more significant periodic acoustic radiation force *F_SSAW_*. In Equation (2), the acoustic contrast factor *ϕ* is what determines the balance position and the acoustically driven direction of the particles in the SSAW field. The particles with positive *ϕ* move towards the pressure node, with the acoustic pressure changing consistently at 0, a particle with negative *ϕ*, and vice versa. Typically, most of the solid particles suspended in an aqueous solution have an instantaneously positive *ϕ* and are concentrated at the pressure node. In the static state of the flow field, the particles will be confined to the pressure node, so that the particles can be captured and arranged. In the flow field state, the acoustic radiation force will attract the particles to move away from the original motion path to the pressure node, so that the focus and separation of the particles can be creatively realized.

**Figure 2:**
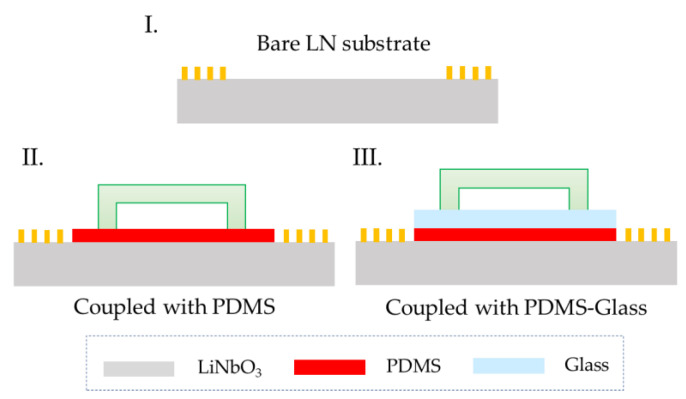
Three device models used for acoustic transmission performance testing.

In our platform, SSAW waves need to pass through the PDMS layer and the glass layer to reach the microchannel, which will have a loss of acoustic waves. For comparison, the three models shown in Figure 2 below were tested: (I) Bare LN substrate, (II) device coupled with PDMS thin film, and (III) device coupled with PDMS–glass thin film.

**Figure 3:**
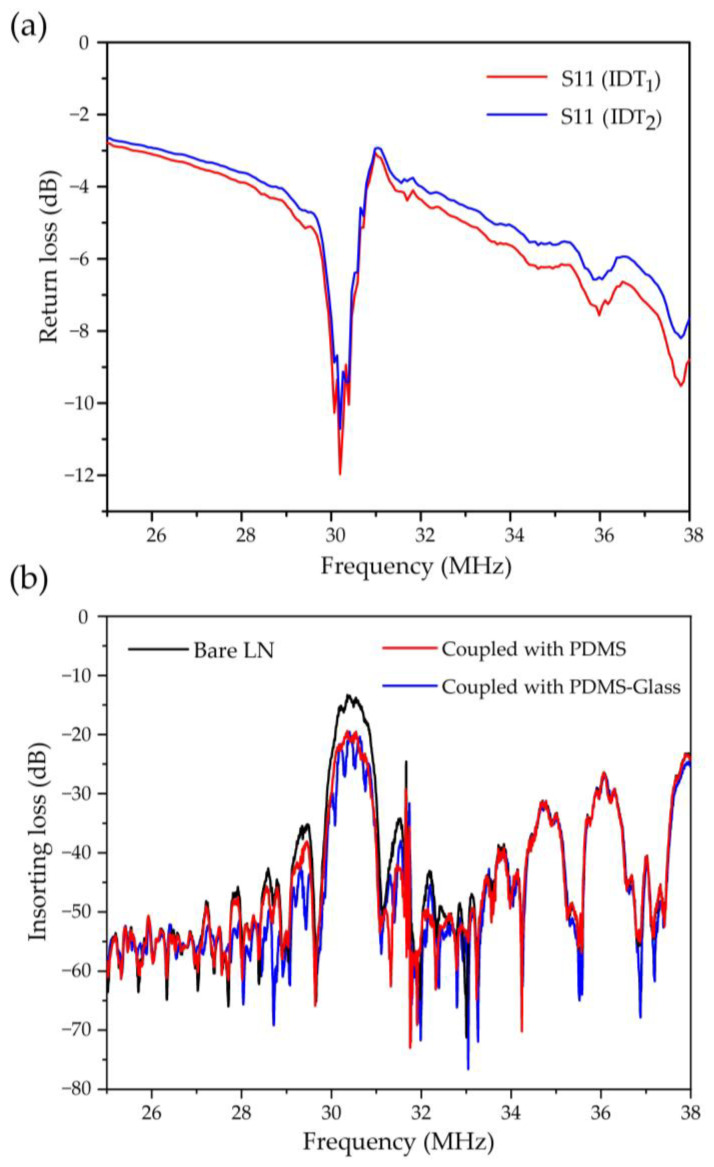
The frequency response of the SAW device: (**a**) the measured return loss (S11) verifies the excitation frequency (30.25 MHz) activated by IDT_1_ and IDT_2_, and (**b**) the measured insertion loss (S21) characterizes the effect of different coupling layers on the acoustic transmission performance.

The measured return loss (S11) (Figure 3a) verified the excitation frequency (30.25 MHz) activated by IDT_1_ and IDT_2_. As shown in Figure 3b, the slight decrease in the measured insertion loss (S21) near the excited frequency (30.25 MHz) was mainly caused by the coupling layer. The results show that the addition of PDMS (red curve) caused only a slight attenuation of acoustic energy, due to its thickness (30 μm) being much smaller than the acoustic wavelength. The addition of PDMS–glass (blue curve) caused almost no acoustic attenuation compared with the PDMS. Experimentally, the platform coupled with PDMS–glass was proven to have a good acoustic transmission capability.

### 2.2. Device Fabrication

**Figure 4:**
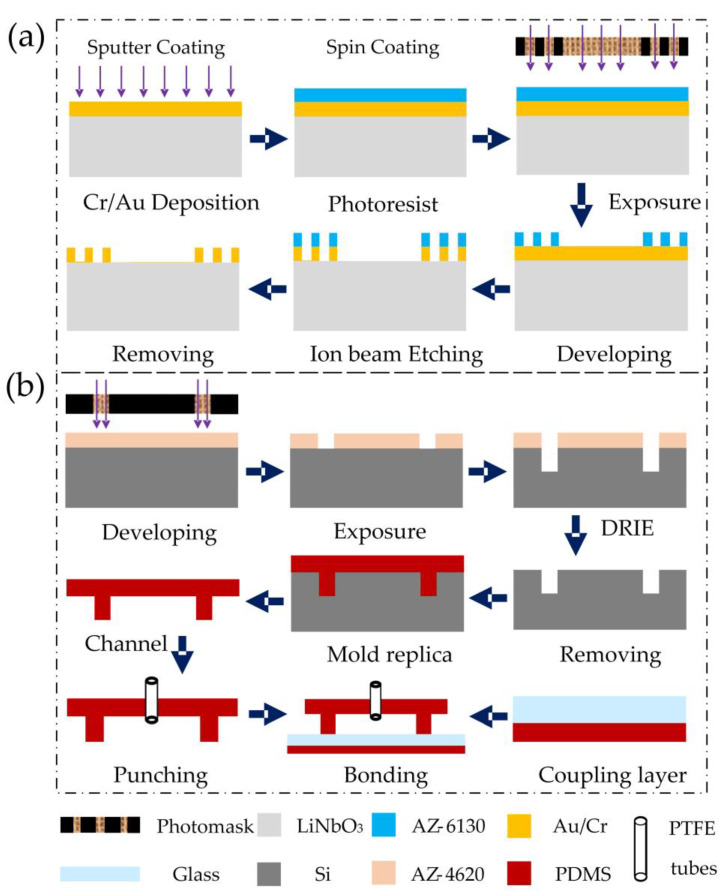
Flow chart of the chip manufacturing process: (**a**) IDT preparation process diagram and (**b**) fabrication schematic of the detachable microchannels.

The reusable SSAW device was composed of a LiNbO_3_ piezoelectric substrate and surface-deposited IDTs. The IDT was designed as a forked finger electrode with a finger width and finger spacing of 30 μm. The IDTs with Cr and Au (20 and 100 nm, respectively) were fabricated by sputter coating and ion beam etching (IBE) on a 500 μm thick, 128° Y-X-axis-rotated cut, X-propagating single crystal LiNbO_3_ substrate. The detailed processes are given in Figure 4a. The detachable channel devices consisted of a 40 μm high PDMS channel bonded to a 130 μm thick glass sheet by O_2_-plasma etching. The PDMS channels were obtained by mold replication, and the channel positive molds were manufactured by photolithography and deep reactive ion etching (DRIE) processes. A 30μm thick PDMS film was adhered to the bottom of the glass plate by spin coating for soft contact with an SSAW device. The film was heated and solidified at 85 °C. Finally, the detachable channel device was lightly pressed onto the SSAW device without any equipment-assisted alignment. The whole process is shown in Figure 4b. The contact remained firm for over 5 days while maintaining a good acoustic transmission performance.

### 2.3. Sample Preparation and System Setup

Reagent 1 was configured with 20 µL of 20 µm polystyrene fluorescent microsphere particle suspension (1 wt%) (20 µm particles show blue under the excitation wavelength of 400 nm), 0.1 mL of Tween 20 (Sevenbio, Beijing, China), and 4 mL of deionized water. Reagent 2 contained 5 µm polystyrene fluorescent microsphere particle suspension (1 wt%) (5 µm particles show green under the excitation wavelength of 488 nm). Reagent 3 was prepared by mixing 2 mL of Reagent 1 and 2, respectively. Reagent 4 was prepared by mixing 2 mL of Reagent 1 and mouse red blood cells (Hongquan Bio, Guangzhou, China), respectively. Different reagents were pumped into the microfluidics chip through a plastic hose (5 mL) with the help of a microinjection pump (XMSP-3C, Ximai, Nanjing, China). The resonant frequency of the SAW device was measured at 30.25 MHz by a vector network analyzer (E5071C, Keysight, Pulau Pinang, Malaysia) and an RF probe station (PW-600, ADVANCED, Taiwan, China). A function signal generator (FY6900-100, FeelElec, Zhengzhou, China) was used to output a sine wave signal of a certain frequency and phase. The particle motion was tracked and recorded using an optical microscope (NIB620-FL, Nexcope, Ningbo, China) and high-speed CCD camera (Nexcam-TC6CCD, Nexcope, Ningbo, China).

## 3. Results and Discussion

### 3.1. Demo I: Parallel Processing and Enrichment of the Sample

The detection and quantification of cell viability and growth commonly involve the harvesting of cells and thus require the parallel alignment of samples for time-lapse or dose–response studies [31]. In this experiment, the capability of a detachable acoustic tweezer platform to process the samples in parallel was designed and demonstrated. A square detachable channel with a length, width, and height of 2.5 × 2.5 × 0.04 mm was set up in the experiment to accommodate large volume samples.

**Figure 5:**
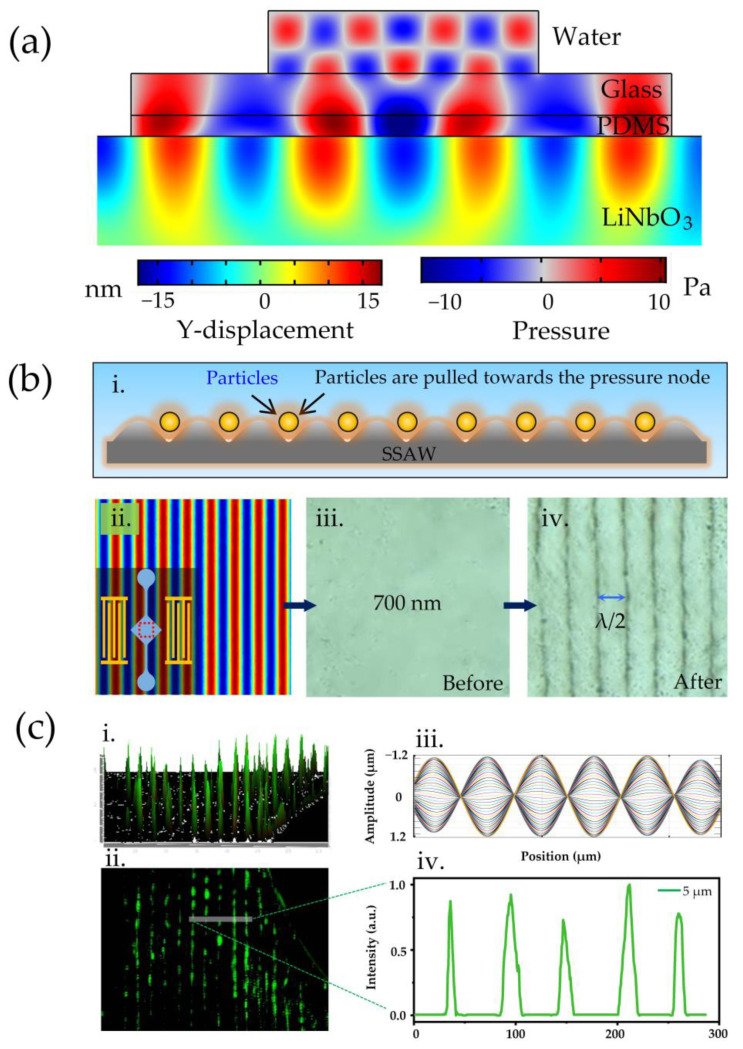
(**a**) Numerical simulation of the wave propagation and radiation on a cross-section of the detachable acoustic tweezer manipulation platform. (**bi**) Schematic diagram of the particle arrangement. (**bii**) Distribution of acoustic pressure. (**biii**,**biv**) Patterning of 700 nm particles before and after SSAW turn-on. (**ci**,**cii**) The aggregation behavior of fluorescent particles observed under the fluorescence field. (**ciii**) The distribution of standing wave amplitude in one cycle. (**civ**) Normalized fluorescence intensity diagram of the arrangement of particles.

Firstly, COMSOL Multiphysics 5.5 was used to simulate the propagation and radiation of the SSAW field. We created a multilayer film model with the same design as in Figure 1c. Two multi-physics fields, the piezoelectric effect and acoustic structure boundary, were used to numerically simulate the vibration displacement of the substrate surface and the internal acoustic pressure distribution of the detachable channel. The simulation results are shown in Figure 5a. Standing wave radiation passed through the waveguide layer with a high acoustic velocity and formed a periodic acoustic pressure field above the glass–water interface. The equilibrium position of the standing wave field was called the pressure node (PN). The particles were pulled towards the equilibrium position by the periodic vibration of the standing wave (Figure 5(bi)). Figure 5(bii) shows the top view of the acoustic pressure field distribution in the channel. Figure 5(biii,iv) indicate the patterning of the 700 nm particles before and after the applied acoustic field, respectively. Furthermore, we demonstrated the light transmission of the detachable manipulation platform. In Figure 5(ci,cii), the aggregation behavior of fluorescent particles observed under the fluorescence field can be observed. Figure 5(ciii) shows the distribution of the standing wave amplitude in one cycle. The arrangement of particles shows a periodic strip distribution (Figure 5(civ) for the normalized fluorescence intensity diagram. Finally, the acoustic retaining ability of the device is verified. After more than 5 days of contact, the particles can still be captured by the acoustic field and achieve a stable arrangement.

### 3.2. Demo II: Capability for Precise Cell Alignment

**Figure 6:**
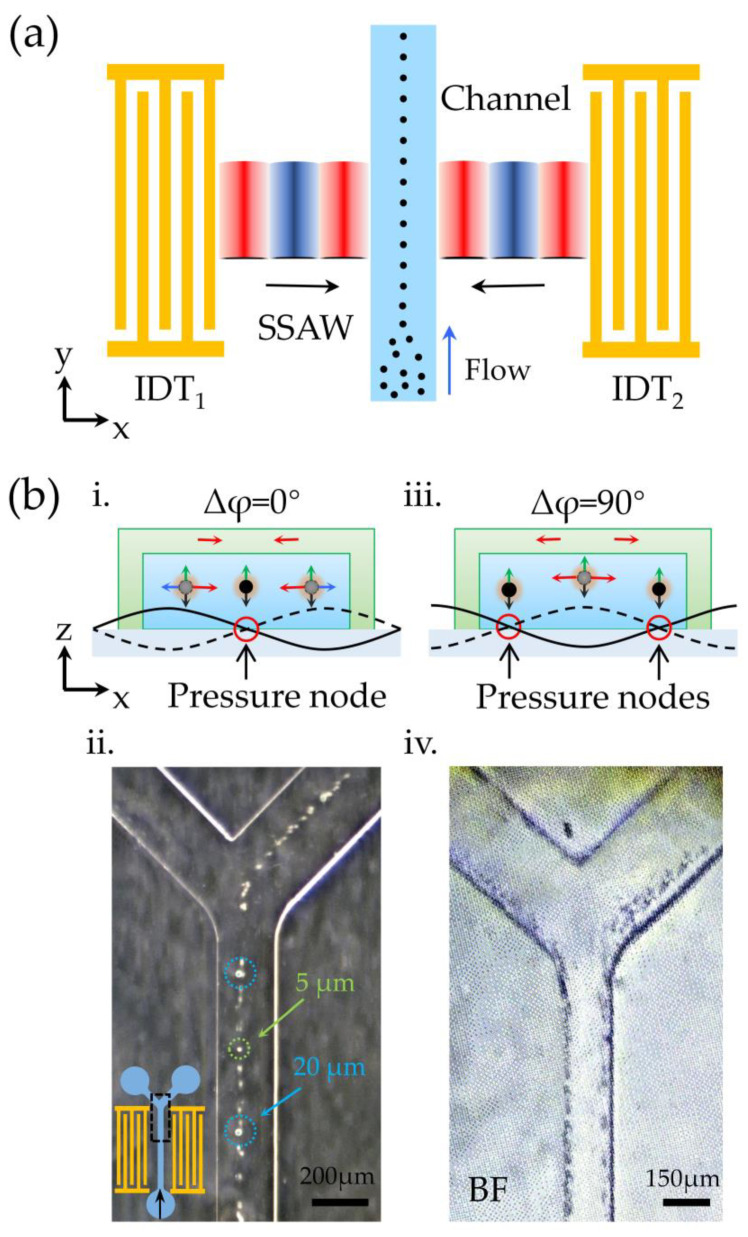
(**a**) Schematic diagram of the particle precision alignment based on the detachable acoustic tweezer platform. (**bi**,**bii**) Force and alignment of the particles in the channel when the pressure line is in the center. (**biii**,**biv**) Force and alignment of particles in the channel when the sound pressure line is on both sides.

In flow cytometry, cells need to be lined up before they pass through the monitoring site [32,33]. Many scholars use hydrodynamic methods to gather particles together [34,35,36]. By adding glass with a high acoustic speed, the acoustic attenuation angle area was smaller. Because of the almost negligible anechoic angle region, no additional sheath flow was needed to align the particles. This is of great significance in flow cytometry detection and counting because the focusing used in flow cytometry requires extra high-speed sheath flow on both sides, and thus damage to cells by shear force is inevitable. The acoustic tweezer manipulation platform provides a method of cell aggregation without sheath flow and without damage. In this experiment, we designed a detachable Y-shaped channel device, the width of which could accommodate only one acoustic pressure line. When the particles entered the SSAW field area, the acoustic radiation force pulled the particles towards the central pressure line and finally lined up into a line. The particle aggregation diagram is shown in Figure 6a. Reagent 3 was pumped into the Y-type detachable microchannel at a flow rate of 0.1 mL/h. We opened the SSAW field and adjusted the phase so that Δφ = 0°, so that the acoustic pressure line was located in the center of the channel (Figure 6(bi)). As a result, particles flowing through the channel were pulled toward the center of the channel and arranged in a neat line (Figure 6(bii)). We adjusted the phase of SSAW so that Δφ = 90°, and the acoustic pressure lines were located on both sides of the channel (Figure 6(biii)). As a result, particles flowing through the channel were pulled to both sides (Figure 6(biv)). This experiment demonstrated the capability of the detachable acoustic tweezer platform to accurately gather cells. It provides a versatile, fast, and relatively inexpensive tool for the highly sensitive detection and counting of flow cytometry.

### 3.3. Demo III: Capability for Separation and Purification of Cells

**Figure 7:**
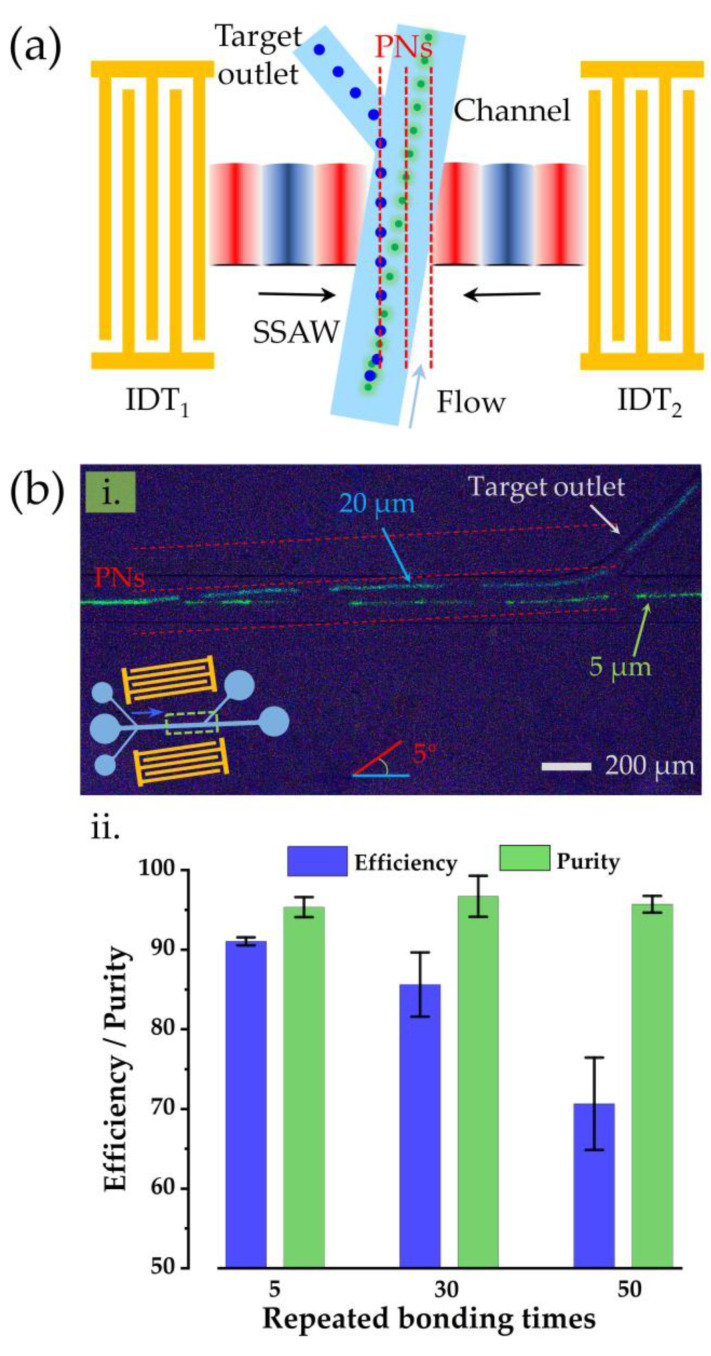
Test results of the device separation performance: (**a**) Schematic diagram of particle separation based on the detachable acoustic tweezer platform. (**bi**) Time-lapse superimposed image of the particle separation process. (**bii**) Performance characterization of the stability and the reusability of the device.

For diluted samples, effective collection by separating the target cells can significantly reduce the amount of fluid handled, for example, the accumulation of trace circulating tumor cells in the human peripheral blood [37,38,39]. The size of the circulating tumor cells in the peripheral blood samples of patients with early-stage cancer is around 14–25 μm [40], while the size of red blood cells with the highest content in the blood samples is about 6 μm [41]. Therefore, Reagent 3 in the sample of this experiment was used to simulate the blood of patients with early cancer, in which 20 μm and 5 μm fluorescent microspheres were used to simulate circulating tumor cells and blood cells, respectively. As a key operation necessary for cell analysis systems, the capability of a detachable acoustic tweezer platform to separate cells was designed and demonstrated. In this experiment, a detachable microchannel with three inlets and two outlets was designed. The cell separation diagram of the device is shown in Figure 7a. When the SSAW was closed, all particles were not subjected to any external forces and flow out of the main channel outlet. When the SSAW was turned on, the larger particles (blue) were subjected to more significant acoustic radiation forces than the smaller particles (green). As a result, the large particles were pushed away from the original streamlines and flowed out of the target outlet along the acoustic pressure line to achieve separation. Figure 7(bi) shows the superimposed image of the time-lapse photo taken by the microscope. The superimposed particle tracks show that the 20 μm particles (blue) moved along the acoustic pressure line (red dotted line), separated from the 5 μm particles (green), and was collected by the target outlet. It can be inferred that this platform has the ability to isolate cancer cells from the blood samples of patients with early-stage cancer.

Finally, the reusability and stability of the detachable acoustic tweezer platform were verified. The experiment was conducted under common conditions. Here, the reusability of the device was defined in terms of the number of repeated adhesives of the channel and the tweezer devices. The separation efficiency and purity of the particles were used to characterize the stability of the device. The separation purity was defined as the percentage of 20 μm particles in the target outlet to the total number of all particles (5 and 20 μm) in that outlet, and the separation efficiency was defined as the percentage of 20 μm particles obtained through separation to the total number of such particles in all of the outlets. The experimental results are shown in the bar chart in Figure 7(bii). The separation efficiency of the particles could be significantly maintained above 92% when the adhesion was repeated five times. When the number of repeated adhesions reached 30 times, the separation efficiency could still be maintained above 85%. After more than 30 adhesive repetitions, the separation efficiency rapidly decayed to only 70%. In the whole process of the experiment, the separation purity of 95% could always be guaranteed. The detachable channel devices could be easily detached from the acoustic tweezer platform after the experiments, allowing the undamaged SSAW device to be reused. This experiment confirms that the detachable acoustic tweezer platform designed in this study had good stability and could be reused up to 30 times or more.

### 3.4. The Actual Application Capability of the Manipulation Platform

**Figure 8:**
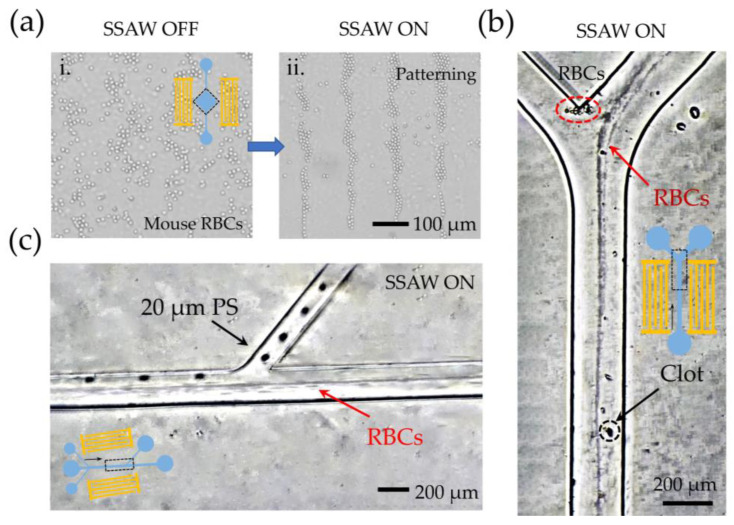
Experimentation on the actual application capability of the manipulation platform: (**a**) the aggregation behavior of RBCs before and after the acoustic field is turned on, (**b**) the precise cell alignment of RBCs, and (**c**) the separation of 20 μm PS particles and red blood cells.

Figure 8a shows the aggregation behavior of RBCs before and after the acoustic field is turned on in ddevice I. The erythrocytes presented a regular patterning under the SSAW field and were still able to maintain their intact cellular morphology, which is very meaningful for the online detection of live cells. Replacing the original channel with channel device II, the RBCs were neatly aligned in a line without the need for additional sheath flow (Figure 8b). This further confirms the practical application capability of our platform for precise cell alignment. Finally, the cell separation function was switched by replacing the channel with detachable device III. Because of sample limitations, 20 μm PS particles were used instead of circulating tumor cells (Reagent 4). The superimposed image (Figure 8c) within a separation step confirmed that large 20 μm particles could be separated from the RBCs, which makes it possible to perform cell separation and purification using our acoustic tweezer platform.

## 4. Conclusions

In this work, we proposed a novel detachable acoustic tweezer manipulation platform. By adding a waveguide layer (glass) and a coupling layer (PDMS polymer film), different detachable microchannel devices could be switched freely from the acoustic tweezer platform. The detachable multi-function acoustic tweezer platform was designed and was demonstrated to be capable of three cell manipulations. In Demo I, it was demonstrated that the detachable acoustic tweezer platform had the capability of parallel individual sample processing. In Demo II, the capability of the platform to accurately align cells was demonstrated, providing a versatile, fast, and inexpensive tool for the highly sensitive detection and counting of the flow cytometry. In Demo III, the capability of the platform to separate and purify cells was demonstrated, and the separation and purification of mixed particles were realized. In addition, the acoustic retention ability, reusability, and stability of the device were verified. The results show that the platform could achieve stable contact for more than 5 days while maintaining a good acoustic transmission performance. The detachable channel devices could be easily detached from the acoustic tweezer platform after the experiments, allowing the undamaged SSAW device to be reused, and the detachable channel devices could be reused 30 times or more. This novel detachable acoustic tweezer manipulation platform enabled the reliable recovery and reuse of acoustic tweezer components, demonstrating the ability to achieve multiple manipulation functions. In the future, it can be further integrated with other on-chip functional units, providing a simple, economical, and multifunctional repeatable platform for biochemical analysis and detection systems.

## Data Availability

Not applicable.
